# Effects of the West Africa Ebola Virus Disease on Health-Care Utilization – A Systematic Review

**DOI:** 10.3389/fpubh.2016.00222

**Published:** 2016-10-10

**Authors:** Kim J. Brolin Ribacke, Dell D. Saulnier, Anneli Eriksson, Johan von Schreeb

**Affiliations:** ^1^Health Systems and Policy Research Group, Department of Public Health Sciences, Karolinska Institutet, Stockholm, Sweden

**Keywords:** indirect health effects, Ebola virus disease, West Africa, systematic review, health systems, infectious disease outbreak

## Abstract

Significant efforts were invested in halting the recent Ebola virus disease outbreak in West Africa. Now, studies are emerging on the magnitude of the indirect health effects of the outbreak in the affected countries, and the aim of this study is to systematically assess the results of these publications. The methodology for this review adhered to the Prisma guidelines for systematic reviews. A total of 3354 articles were identified for screening, and while 117 articles were read in full, 22 studies were included in the final review. Utilization of maternal health services decreased during the outbreak. The number of cesarean sections and facility-based deliveries declined and followed a similar pattern in Guinea, Liberia, and Sierra Leone. A change in the utilization of antenatal and postnatal care and family planning services was also seen, as well as a drop in utilization of children’s health services, especially in terms of vaccination coverage. In addition, the uptake of HIV/AIDS and malaria services, general hospital admissions, and major surgeries decreased as well. Interestingly, it was the uptake of health service provision by the population that decreased, rather than the volume of health service provision. Estimates from the various studies suggest that non-Ebola morbidity and mortality have increased after the onset of the outbreak in Sierra Leone, Guinea, and Liberia. Reproductive, maternal, and child health services were especially affected, and the decrease in facility deliveries, cesarean sections, and volume of antenatal and postnatal care visits might have significant adverse effects on maternal and newborn health. The impact of Ebola stretches far beyond Ebola cases and deaths. This review indicates that indirect health service effects are substantial and both short and long term, and highlights the importance of support to maintain routine health service delivery and the maintenance of vaccination programs as well as preventative and curative malaria programs, both in general but especially in times of a disaster.

## Introduction

On March 29, 2016, the WHO Director-General terminated the Public Health Emergency of International Concern for the Ebola virus disease (EVD), an outbreak of hemorrhagic fever in West Africa. By then, an estimated 30,000 people had been infected with EVD, and over 10,000 people had died as a direct consequence, the majority in Guinea, Liberia, or Sierra Leone (1).

In December 2013, the index Ebola case in the West Africa outbreak was infected, and the disease was identified as Ebola in March 2014 (2). During the next year, the number of confirmed Ebola cases and deaths in the three most affected countries increased weekly, until joint national and international efforts to stop the epidemic finally led to its gradual decline by the second half of 2015. Before the outbreak, health indicators in West Africa were among the worst in the world, particularly in terms of maternal and child health (3). In this fragile context, the outbreak was a significant shock to the health systems and led to fear and mistrust of the health services, the closure of health facilities, and the deaths of health service staff (4). During the outbreak, health workers were 21–32 times more likely to be infected with Ebola than the general adult population (5). While the international response was considerable – hundreds of Ebola Treatment Units were built – it came late and focused mainly on isolating EVD cases, thereby leaving the treatment and provision of services for the regular burden of disease and routine health care largely unattended (6). For example, previous studies completed by the authors who assessed nationwide routine health service delivery data during the EVD epidemic in Sierra Leone found that hospital functioning was significantly reduced during the outbreak. This information suggests that the indirect effects of EVD on health system functioning might have adversely affected more people than the virus itself (7, 8).

Studies on the indirect health effects of the EVD outbreak are now starting to emerge. While the studies all add important pieces of information, few of them were done at a nationwide level and even fewer covered the whole affected area. Therefore, a systematic review is important at this early stage in order to create a comprehensive picture. By outlining the evidence for what affects the outbreak had on health, it may be possible to help guide policy on health system strengthening and develop resilience for withstanding future shocks. The aim of this study is to assess the magnitude of indirect health effects of the West Africa EVD outbreak, based on existing literature.

## Materials and Methods

The search strategy for the review was developed in collaboration with librarians at the Karolinska Institutet university library. After several pilot searches, the main search was completed in March 2016 and included the following databases: Medline (Ovid), Embase, Web of Science, Global Health (Ovid), POPline, and PubMed. Keywords used in the search included variations in terms for Ebola, health, health systems, health facility, and illness/disease. Relevant gray literature was identified from the various actors on-site in the affected countries during the outbreak. WHOLIS (WHO plus iris), WorldCat Libraries, and Base Bielefeld databases were also searched for gray literature. Full documentation of the search strategy can be found in Supplement 1. The study was registered on PROSPERO under registration number CRD42016036318 (9).

The focus of the review is the West Africa Ebola outbreak, due to its unprecedented magnitude. The affected population was defined as persons residing in Guinea, Liberia, or Sierra Leone, or in the affected areas of Nigeria, Senegal, and Mali. Studies from countries with three or less cases of EVD were not included, since the indirect impact of a small number of cases is likely to be negligible. Direct health effects were defined as morbidity and mortality due to infection with the Ebola virus. Indirect effects refers to changes in health outcomes caused by the effect of the West Africa EVD outbreak on the health systems, including but not limited to increased maternal morbidity and mortality, a reduction in HIV-infected patients receiving antiretroviral treatment (ARTs), an increase in malaria cases due to termination of intermittent preventative treatment (IPT) programs, fewer children being treated for diarrhea and acute respiratory infections (ARI) and hospital in-patient admittance and essential surgery.

Because of the scarcity of literature currently available on the indirect effects of the Ebola outbreak, the eligibility criteria were deliberately broad. Studies were included in the analysis if they contained information on indirect health effects of EVD and had either an abstract or full-text available in the English language. Studies were excluded if they reported information only on the direct effects of EVD, if mental health was the outcome under study, or if the publication was a commentary article. Gray literature and several reports were included in the review; all reports were assessed for potential bias regarding the goals of the author organization. An inclusive approach was taken, and several modeling studies are therefore included in the analysis.

**Figure 1:**
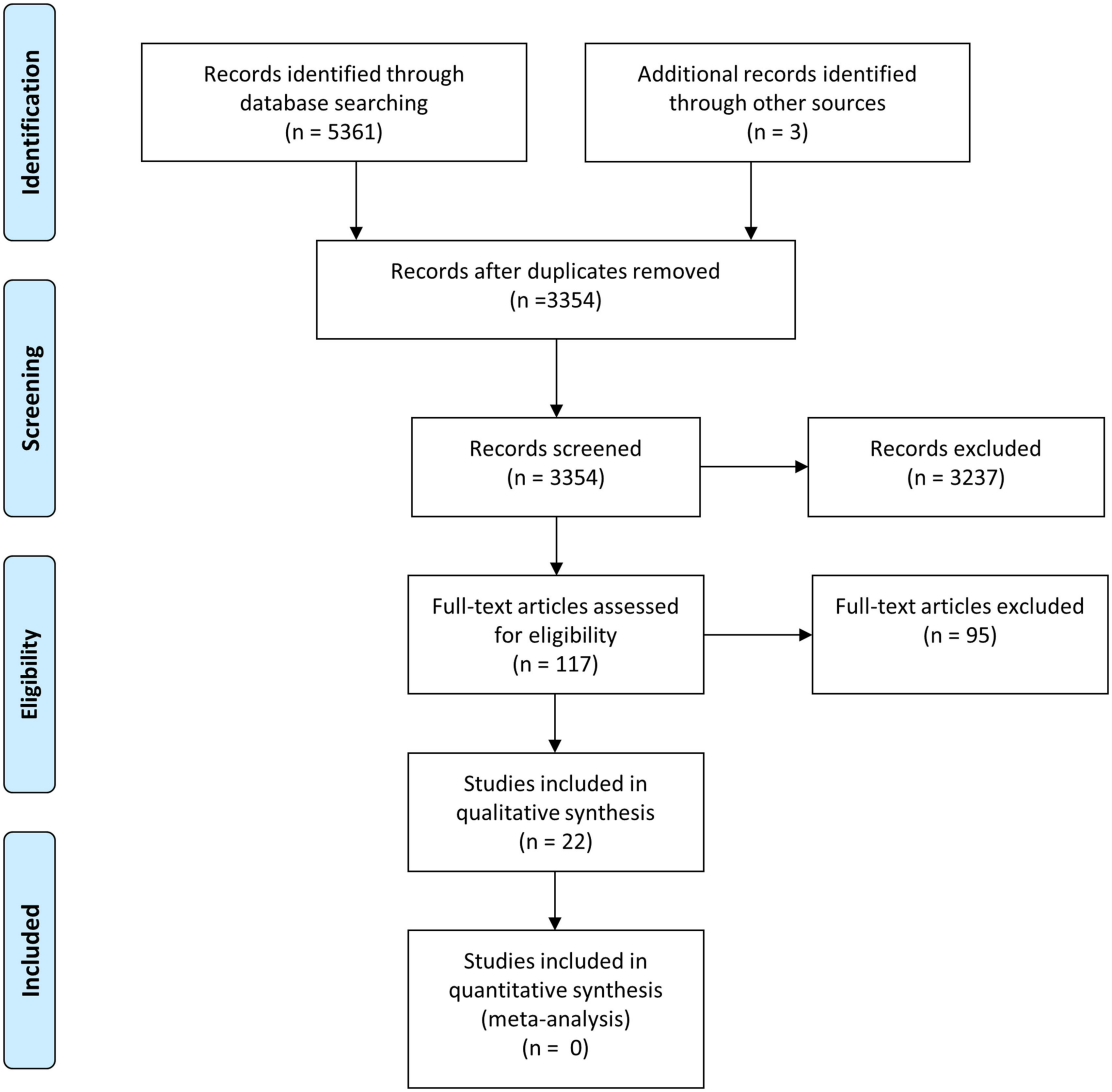
**Prisma flow diagram of search procedure and screening process**. From Moher et al. (10). For more information, visit www.prisma-statement.org.

The searches produced 3354 articles after deduplication. A total of 2788 articles were excluded during title screening and 442 were excluded after abstract screening. About 117 articles were read in full, and 22 studies were included in the final review. Other relevant studies were identified for inclusion through the reference lists of included studies. Figure 1 is showing a Prisma diagram of the search and screening procedure. Two of the authors performed the screening process independently, and in case of uncertainty about inclusion, a consensus was reached among the authors after discussion. All articles were organized in an EndNote X7 library.

Information on location of the study, the objective, the study design, the outcome(s) under study, and results was extracted into a spreadsheet file for analysis. After extraction, information was grouped by outcome for the analysis, resulting in three broad categories: reproductive, maternal, and child health services (RMNCH); HIV/AIDS, tuberculosis or malaria; and other outcomes, such as cause of death. The evidence was rated for quality, using the GRADE approach as a guide, and divided into one of the four quality levels: high, moderate, low, and very low (11). In addition to study quality, the relevance of each study to the aim of the review was assessed.

## Results

The review included 22 studies, of which 19 were peer-reviewed scientific papers or policy briefs. The remaining three were reports by UNICEF, Doctors of the World, and Voluntary Services Overseas. Eight of the studies focused on Sierra Leone, six on Guinea, and three on Liberia. The remaining five studies included all three countries. Key health indicators for each country can be found in Table 1. A summary of the studies can be found in Table 2.

**Table 1:** **Key health indicators**.

Country	U5 mortality (per 1000 live births)	Maternal mortality ratio (per 100,000 live births)	Medical Doctors (nationwide)	Nurses, midwives (nationwide)	Health-care workers per 10,000 inhabitants
Liberia	112	725	150	1000	3
Sierra Leone	192	1360	200	1000	2
Guinea	142	679	1200	4400	5

*Reference: The 2014 update, Global Health Workforce Statistics, World Health Organization, Geneva (http://www.who.int/hrh/statistics/hwfstats/). Reservation for time lag*.

**Table 2:** **Results per study**.

Reference	Location of study	Study setting, data source, and comparative dates	Study objective	Main findings	Quality (Q) and relevance (R)
Barden-O’Fallon et al. (12)	Guinea	Convenience sample of 16 hospitals (H) and 29 health centers (HC)	To understand how the delivery and utilization of routine RMNCH services had been affected by the Ebola outbreak	– Decrease in outpatient visits: 31% (H), 6% (HC), *p* < 0.001. – Maternal indicators; only HIV testing in pregnant women showed significant decrease: 51% (H), *p* < 0.05. – Pentavalent vaccine dose 1 and 3 decrease at HC: −18%/−32%, *p* < 0.001 (at H, no sign change). – Decrease in children seen for diarrhea: 60% (H), 25% (HC), *p* < 0.001. – Decrease in children seen for ARI: 58% (H), 23% (HC), *p* < 0.001.	Q: Moderate
	12 prefectures (“active” and “inactive” for Ebola)	Routine facility records			R: High
		October to December 2013 to October to December 2014			
Bolkan et al. (7)	Sierra Leone	All health facilities providing surgical care	To quantify to what extent admission rates and surgery changed at health facilities during the Ebola outbreak	– 70% decrease in median number of admissions (*p* = 0.005) between May and October 2014. – 50% decrease in number of major surgeries (*p* = 0.014) between May and October 2014.	Q: Moderate
	Countrywide	Routine admission and surgical theater register books			R: High
		January to September 2014 to September to October 2014			
Brolin Ribacke et al. (8)	Sierra Leone	All health facilities providing emergency obstetric care	To assess the potential impact of Ebola on nationwide access to obstetric care	– Decrease in in-hospital deliveries: 21% (p2), −28% (p3), *p* < 0.05 – Decrease in volume of cesarean sections: 22% (p2), 20% (p3), *p* < 0.05 *Change per sector*: – Governmental: deliveries: −15% (p2), −36% (p3). Cesarean sections: −5% (p2), + 5% (p3). – Private non-profit: deliveries: −37% (p2), −5% (p3). Cesarean sections: −49% (p2), −58% (p3).	Q: Moderate
	Countrywide	Routine facility records			R: High
		January to May 2014 (p1), May to December 2014 (p2), January to May 2015 (p3)			
Bundu et al. (13)	Sierra Leone	Single surgical institution	To detail the effect of the Ebola outbreak on a surgical institution	– By August 2014: accident and emergency (A&E) presentations and ward admissions had fallen to: A&E 120 vs. 51; ward admissions 100 vs. 51. – By December 2014: A&E admissions from 115 to 42 (37%) and ward admissions from 147 to 23 (16%). Operative surgery: by August 2014:19% of 2013 level. By December 2014 (after deaths of 2 out of 8 surgeons): 3% of 2013 level.	Q: Low
	Freetown, Western Urban district	Routine facility records			R: Medium
		June 2013 to May 2014 to May 2014 to February 2015			
Cisse et al. (14)	Guinea	Single national hospital	To assess the impact of the Ebola outbreak on the quality of care of PLHIV taking ART	– Number of visits in clinic/pharmacy decreased from 3062 in April to 2794 in June. – When length of time *x* = 90 days, the proportion of defaulters increased from 1% in April to 15% in June, from 2 to 22% when *x* = 80 days and from 4 to 34% when *x* = 70 days. (Poster presentation)	Q: Low
	Conakry, Conakry region	Routine prescription records			R: Medium
		January to June 2014			
Dynes et al. (15)	Sierra Leone	Six primary health-care facilities	To assess attitudes and perceptions regarding the risk of Ebola and health facility use among health workers and pregnant and lactating women	– Number of first antenatal care visits in the district decreased by 29%, from 2086 in May to 1488 in July (2014). – Number of postnatal care visits within 48 h after delivery decreased by 21%, from 1923 in May to 1512 in July. – Consensus among facility staff and pregnant and lactating women that the primary reason for decreased use of health facilities was fear of contracting Ebola at a facility, including outpatient facilities.	Q: Moderate
	Kenema district	Focus group discussions with health workers, support staff, and pregnant or lactating mothers			R: Medium
		May to July 2014			
Elston et al. (16)	Sierra Leone	15 health facilities	To assess the impact of the Ebola outbreak on health systems	– CHC attendance in Lower Banta Chiefdom: reduced during October 2013 to January 2014 (pre-Ebola) compared with the equivalent period peri-Ebola outbreak, *p* = 0.025. – CHC attendance in Ribbi Chiefdom decreased during October 2013 to January 2014 (pre-Ebola) compared to equivalent period peri-Ebola outbreak, *p* < 0.001. – In Lower Banta among women aged ≥15 years, 324 women attending the CHC in November 2013 for urgent, routine, and antenatal care compared with just one person in November 2014. – Deliveries in health facilities reduced particularly in the most Ebola-affected areas (*p* = 0.017).	Q: Low
Moyamba and Koinadugu districts	Facility records, district health records; focus group discussions and interviews with health workers and burial teams	R: Medium		
	August to December 2013 to August to December 2014			
Evans et al. (17)	Guinea, Liberia, and Sierra Leone	–	To estimate how loss of health-care workers will impact non-Ebola deaths in the future	Modeling paper: – Maternal mortality to increase by 38% (G), 74% (SL), 111% (L); 4022 additional deaths per year. – Infant mortality to increase by 7% (G), 13% (SL), 20% (L); 6700 additional deaths per year. – U5 mortality to increase by 10% (G), 19% (SL), 28% (L); 14,100 additional deaths per year. – Aggregated: estimated 24,900 additional deaths per year.	Q: Moderate
		WHO and World Bank indicators			R: Medium
		May 2014 to May 2014			
Hyjazi et al. (18)	Guinea	–	To assess the impact of the Ebola epidemic on the utilization of maternal and reproductive health-care services	– Delivery care dropped 81% (*n* = 2490 to *n* = 463) in N’zérékoré and 74% (*n* = 4890 to *n* = 1724) in Conakry between Q1 and Q5. – Ceasarean sections also declined sharply. – Delivery care unchanged in Kankan and Faranah regions until Q5. – Family planning services: average monthly users fell 75% in N’zérékoré (*n* = 10,703 to *n* = 2580), 53% in Conakry (*n* = 6191 to *n* = 2893) and 65% in Kankan (*n* = 11,660 to *n* = 4082). (Abstract only)	Q: Low (abstract only)
	3 eastern regions and Conakry	Health systems strengthening project data			R: Medium
		October 2013 to December 2014 (quarterly comparisons)			
Helleringer and Noymer (19)	Guinea, Liberia, and Sierra Leone	–	To assess the impact of the Ebola outbreak on the utilization of selected maternal and newborn health services	Modeling paper: – Liberia: EVD deaths exceeded the expected number of deaths due to the leading non-EVD cause of death. – Sierra Leone: EVD might have killed more people in 2014 than the leading non-EVD cause of death (i.e., malaria). In other sets of model parameters, EVD still killed more people than the second (i.e., lower respiratory infections) or the third (i.e., HIV/AIDS) leading causes of death. – Guinea: EVD never ranked higher than the top three non-EVD causes of death.	Q: Moderate
		National reported EVD data			R: Medium
		2014			
Iyengar et al. (20)	Liberia	All primary health facilities in counties	To compare EVD with other causes of death	– Data show a decrease in absolute utilization from the start of the outbreak, followed by a slow recovery after October or November. – In Bong County, antenatal care (ANC) visits and intermittent preventative treatment in pregnancy (IPTp): less than 14% of the peak numbers during the outbreak. – Total deliveries, utilization less than 33% of the highest month. – In Margibi County, during height of the outbreak, numbers less than 9% of peak utilization for ANC visits and 4% for IPTp. – Total health facility deliveries less than 9% of peak utilization.	Q: Moderate
	Margibi and Bong counties	District health information database			R: Medium
		March 2014 to December 2014			
Leuenberger et al. (21)	Guinea	Single HIV care facility	To investigate the impact of Ebola on general and HIV care	– Throughout 2014, service offer was continuous and unaltered at the facility. – During August to December 2014 compared with the same period of 2013 attendance at the primary care outpatient clinic (−40%), HIV tests done (−46%), new diagnoses of tuberculosis (−53%) patients enrolled into HIV care (−47%). Reduction in attendance at the HIV follow-up clinic (−11%).	Q: Moderate
	Macenta district, Nzérékoré region	Routine facility data			R: High
		August to December 2013 to August to December 2014			
Lori et al. (22)	Liberia	12 health facilities	To examine the influence of Ebola on the use of facility-based maternity care	Prior the EVD outbreak, facility-based deliveries steadily increased in Bong County reaching an all-time high of over 500 per month at study sites in the first half of 2014 – indicating Liberia was making inroads in normalizing institutional maternal health care. However, as reports of EVD escalated, facility-based deliveries decreased to a low of 113 in August 2014	Q: Moderate
	Bong county	Routine facility records			R: High
		January 2012 to October 2014			
Loubet et al. (23)	Liberia	Two HIV care clinics	To assess the potential effect of the epidemic on the care of HIV patients	– From June 2014, the number of visits per week, stable since 2012, abruptly decreased (59%) in Redemption H (*p* < 0.001) and progressively decreased by 3% per week in JFK H (*p* < 0.001). – In both clinics, the weekly proportion of patient with follow-up delay sharply increased after the point break from June 2014 (*p* < 0.001). – From June 2014, a significant decrease in new patients per week occurred in both the clinics: by 57% (*p* < 0.001) in Redemption H and by 4.6% per week (*p* < 0.001) in JFK H.	Q: High
	Monrovia, Montserrado district	Routine facility data			R: Medium
		January 2012 to June 2014 to June 2014 to October 2014			
Ndawinz et al. (24)	Guinea	HIV facility at a single hospital	To determine the true effect of the Ebola epidemic on the continuum of HIV care	– From April to December 2014, the proportion of defaulters among patients receiving ART increased from 0% to 42% (*p* < 0.0001). – The number of patients active in care decreased between June and December (*p* < 0.05). – Risk of default was highest between June and September 2014, while the Ebola epidemic was increasing exponentially.	Q: Low
	Conakry, Conakry region	Routine facility data			R: Medium
		2014			
Parpia et al. (25)	Guinea, Liberia, and Sierra Leone	–	To estimate the repercussions of the Ebola outbreak on the populations at risk for malaria, HIV/AIDS, and tuberculosis	Modeling paper: – A 50% reduction in access to health-care services during the Ebola outbreak exacerbated malaria, HIV/AIDS, and tuberculosis mortality rates by additional death counts of 6269 (2564–12,407) in Guinea; 1535 (522–28,780) in Liberia; and 2819 (844–4844) in Sierra Leone	Q: Moderate
		WHO, DHS, and Global Burden of Disease reports			R: High
		March 2014 to March 2015			
Plucinski et al. (26)	Guinea	120 public health facilities	To characterize malaria case management in the context of the Ebola epidemic and document the effect of the outbreak on malaria case management	– Substantial reductions in all-cause outpatient visits (11%), cases of fever (15%), and patients treated with oral (24%) and injectable (30%) antimalarial drugs in surveyed health facilities. – In Ebola-affected prefectures, 73 of 98 interviewed community health workers were operational (74%, 95% CI 65–83) and 35 of 73 were actively treating malaria cases (48%, 36–60) compared with 106 of 112 (95%, 89–98) and 102 of 106 (96%, 91–99), respectively, in Ebola-unaffected prefectures. – Nationwide, the Ebola virus disease epidemic was estimated to have resulted in 74,000 (71,000–77,000) fewer malaria cases seen at health facilities in 2014.	Q: Moderate
	4 most affected prefectures, 4 randomly selected unaffected prefectures	Cross-sectional survey on malaria case management; registry indicators, national surveillance data			R: High
		March 2013 to March 2015			
Takahashi et al. (27)	Guinea, Liberia, and Sierra Leone	–	To understand how Ebola-related health-care disruptions increases the risk from measles	Modeling paper: – Estimate that at the start of the Ebola crisis, there were 778,000 [95% credible interval (CrI): 715,000 to 915,000] unvaccinated children in the three countries. – With every month of health-care disruptions, estimate the number of children between 9 months and 5 years of age who are not vaccinated against measles increases by an average of 19,514 (assuming a 75% reduction in vaccination rates nationally), reaching 964,346 (95% CrI: 862,682–1,129,026) after 6 months, 1,068,833 (95% CrI: 914,108–1,288,857) after 12 months, and 1,129,376 (95% CrI: 934,926–1,409,052) after 18 months.	Q: High
		DHS data			R: High
		Estimated 6, 12, or 18 months of health service disruption			
Walker et al. (28)	Guinea, Liberia, and Sierra Leone	–	To quantify the additional indirect burden of Ebola on health systems and malaria care and control	Modeling paper: – If malaria care ceased as a result of the Ebola epidemic, untreated cases of malaria would have increased by 45% (95% credible interval 43–49) in Guinea, 88% (83–93) in Sierra Leone, and 140% (135–147) in Liberia in 2014. – This increase is equivalent to 3.5 million (95% credible interval 2.6 million to 4.9 million) additional untreated cases, with 10,900 (5700–21,400) additional malaria-attributable deaths. – Mass drug administration and distribution of insecticide-treated bed nets timed to coincide with the 2015 malaria transmission season could largely mitigate the effect of Ebola virus disease on malaria.	Q: Moderate
		DHS data			R: High
		Through November 2014			
VSO International (29)	Sierra Leone	All facilities providing complex and basic emergency obstetric care	To determine the impact of Ebola on the provision of maternal and newborn care	– Nationwide decrease: −11% facility deliveries. ANC, PNC visits: significant decrease for 6/13 districts. – May 14-September 14: deliveries −31% BEmOCs, −37% CEmOCs. – May 14-January 15: deliveries −63% CEmOCs. In 8 districts: PNC visits: −22%. ANC visits: −18%. – 30% rise in maternal CFR across all facilities, significant in CEmOCs [IRR: 1.44 (1.17–1.75)]. – 24% increase in incidence of stillbirth, significant in CEmOCs [IRR: 1.27 (1.16–1.39)]. – Signal functions: improved availability of removal of retained products of conception and neonatal resuscitation. Deterioration of availability of assisted vaginal del at BEm.OCs.	Q: Low
	Countrywide	Routine facility data; interviews			R: Medium
		May 2013 to April 2014 to May 2014 to April 2015			
UNICEF (30)	Sierra Leone	All primary health facilities nationwide	To evaluate the extent to which utilization of key maternal and child health interventions could be maintained during the Ebola outbreak	Nationwide decreases: – ANC4 visits: −27%. – ITNs distribution at ANC vistis: −63%. – Facility deliveries: −27%. – Children receiving Penta3: −21%. – U5 receiving malaria treatment: −39%. – PMTCT: −23%.	Q: Moderate
	Countrywide	Routine facility data			R: High
		May 2014 to September 2014			
Doctors of the World (31)	Sierra Leone	Primary health facilities in Moyamba district	To identify and describe the current state of the health system and priority health issues affecting the population	– Attendance decreased by 73/52/21/0% across the district. – Antenatal attendance decreased by 50% and deliveries decreased by 43% at Bradford hospital. – Maternity admissions decreased at MGH and pediatric admissions decreased by 75% at MGH. Pediatric admissions due to malaria: −80% at MGH. Outpatient consultations: −67%. – Number of fully vaccinated children decreased by 26% at Moyamba CHC.	Q: Low
	Moyamba district	Routine facility data; focus group discussions and interviews with local stakeholders, health-care workers, and patients			R: Medium
		October 2013 to January 2014 to October 2014 to January 2015			

**Figure 2:**
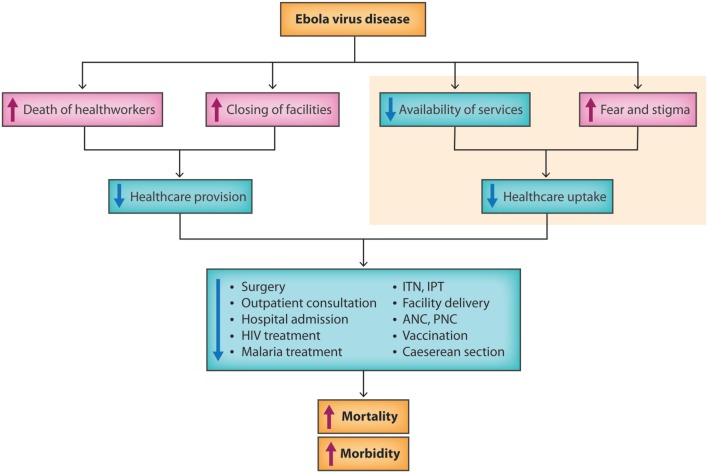
**A flow chart of the indirect health effects of the West Africa EVD outbreak**. Illustration by Dr. Sandra Bark.

Eleven studies investigated how EVD impacted RMNCH, 10 focused on HIV/AIDS, tuberculosis, or malaria, and 4 reported other outcomes of changes in patient admissions and surgery and causes of death. There was some overlap within the different health outcome categories in the studies. The results of each category are described below. The main results of this review are visualized in Figure 2, which aims to provide an overview of the indirect effects the EVD outbreak had on health and health-care utilization.

### Reproductive, Maternal, and Child Health

The utilization of maternal health services decreased during the outbreak. The number of cesarean sections and facility-based deliveries declined and followed a similar pattern in all three countries. In Sierra Leone, a nationwide decrease of 20% (*p*-value <0.05) for cesarean sections and facility deliveries at surgical facilities was seen during the outbreak (8). Also in Sierra Leone, two studies including primary health facilities and facilities offering basic emergency obstetric care saw facility-based deliveries dropping by 27–37% during the second half of 2014 (29, 30). In Moyamba and Koinadugu districts, two districts heavily affected by EVD, two studies found a decline in facility-based deliveries of over 50% during the second half of 2014, compared to the second half of 2013 (16, 31). In Liberia, the number of facility-based deliveries had dropped to 9% of peak utilization before the outbreak in one county (20), while the second single county saw a brief rise in deliveries in the first half of 2014, followed by a decline to 113–306 deliveries per month during August to October compared to the average of 400–500 during the first 7 months of 2014 (22). In Guinea, a sharp decline of 74–81% in delivery care occurred in two of the regions most affected by EVD during the last quarter of 2014 (18).

A change in the utilization of antenatal (ANC) and postnatal care (PNC) and family planning services was also seen. Across Sierra Leone, ANC and PNC visits decreased: nationwide during the last 6 months of 2014, visits dropped significantly in 6 out of 14 districts (29) and the number of women’s fourth ANC visit dropped by 27% (30). In Moyamba district, antenatal attendance decreased by over 50% at a hospital during the same time period (31), and in Kenema district, the number of first ANC visits dropped by 29% and the number of PNC visits in the first 48 h after delivery fell by 21% between May and July 2014 (15). In Liberia, ANC visits dropped to 9–14% of pre-EVD peak numbers in two districts during the last 6 months of 2014 (20). While no studies on changes in ANC or PNC in Guinea were found, two studies reported on the utilization of family planning services: no significant change in utilization was seen in a convenience sample of public and private health facilities across the country during the last 3 months of 2014 (although a significant decrease of 51% was seen in HIV testing at the hospital level, *p* < 0.05) (12), while analysis of routine health service data in three regions did see a decline of 50–75% in family planning services over the course of the outbreak (18).

The drop in utilization applied to children’s health services as well. In Guinea, fewer children under 5 years (U5) were seen at hospitals and health centers for diarrhea [60% decrease at hospitals, 25% decrease at health centers (*p* < 0.001)] and ARI [58% decrease at hospitals, 23% decrease at health centers (*p* < 0.001)] over the course of 2014 (12). In regard to vaccination, the distribution of pentavalent vaccine doses 1 and 3 significantly decreased at health centers in Guinea by 18–32% (*p* < 0.001) (12), the number of children receiving dose 3 of pentavalent vaccine decreased by 21% across Sierra Leone (30), and a hospital in Moyamba district saw a 26% decrease in fully vaccinated children (31).

A modeling study for all three countries investigated how a 6-, 12-, or 18-month disruption of health service provision would affect the risk of measles in children. An estimated 778,000 children between the ages of 9 months and 5 years in these countries were unvaccinated before the EVD outbreak. Every additional month of health service disruption would result in approximately 20,000 more unvaccinated children, amounting to 1.5 million unvaccinated children at 18 months. In this scenario, the likely number of measles cases during an outbreak would double from 100,000 to 200,000 (27).

In terms of mortality, there was limited information. A significant rise was seen in comprehensive emergency obstetric and newborn care facilities (CEmONCs) across Sierra Leone for the maternal case fatality rate [incidence rate ratio (IRR): 1.44, 95% CI: 1.17, 1.75] and for stillbirths (IRR: 1.37, 95% CI: 1.16, 1.39) (29). A modeling paper assessed how death of health-care workers would impact non-Ebola deaths in the future across all three countries. The results estimated that maternal mortality would increase by 28% in Guinea, 74% in Sierra Leone, and 111% in Liberia, adding up to an additional 4022 maternal deaths per year. The second modeled scenario found an increase of 7% for infant mortality and 10% for U5 mortality in Guinea, 13% infant and 19% U5 in Sierra Leone, and 20% infant and 28% U5 in Liberia, or an additional 6700 infant deaths and 14,100 U5 deaths per year. Combined, these numbers amount to 24,900 additional deaths per year due to the death of health-care workers during the EVD outbreak (12, 15, 17, 32).

### HIV/AIDS and Malaria

As seen with RMNCH, the uptake of HIV/AIDS and malaria services decreased as well. For HIV/AIDS services overall, the number of patients visiting facilities for HIV care significantly decreased in Guinea during the last 6 months of 2014 (*p* < 0.05) (24) and Liberia between June and October of 2014 (*p* < 0.001) (23). The prevention of mother-to-child transfer of HIV in Sierra Leone decreased by 23% during mid-2014 (30). The diagnosis and treatment of new HIV patients declined at HIV treatment facilities in both Guinea (a 46% drop in HIV testing and a 47% in enrollment of new HIV patients in treatment) (21) and in Liberia (a cumulative decrease of 57% at one clinic and a 4.6% decrease per week at a second clinic, *p* < 0.001) (23). Two studies from Guinea found an increase in the number of defaults in renewing ART prescriptions, which corresponded with the increase in EVD cases (14, 24). The number of patients with a delay of at least 3 months for follow-up HIV care sharply increased (*p* < 0.001) between June and October in Liberia (23).

For malaria cases, a decrease of 15% in cases of fever seen at health facilities was noted in Guinea, leading to an estimation that 74,000 fewer cases of malaria were seen between March 2013 and March 2015 (26) and pediatric admissions for malaria fell by 80% at a hospital in Sierra Leone after the onset of the Ebola outbreak (31). For malaria services, a general decrease in treatment services was seen. Treatment with antimalarial drugs decreased by 24–30% at health facilities in Guinea (26), IPT during pregnancy dropped to 4–14% of pre-Ebola peak numbers in Liberia (20), and the number of children under 5 years of age receiving malaria treatment declined by 39% during the outbreak in Sierra Leone (30).

Two modeling studies took a different approach in estimating the indirect impact of Ebola on mortality and service delivery. One study estimated the expected change in malaria cases and deaths across all three countries during the outbreak by removing the effect of treatment and hospital care, and then modeled the potential effect of emergency malaria interventions. If malaria care had ceased, untreated cases of malaria would have increased by 45% in Guinea, 88% in Sierra Leone, and 140% in Liberia, or a total of up to 3.5 million additional untreated malaria cases and 10,900 additional malaria deaths. However, starting mass drug administration and distribution of insecticide-treated nets could mitigate the effect of the outbreak on malaria (28). The second study using a computational model estimated that a 50% reduction in access to health care during the EVD outbreak would exacerbate the number of deaths from malaria, HIV/AIDS, and tuberculosis by approximately 6269 excess deaths in Guinea, 1535 in Liberia, and 2819 in Sierra Leone. The estimates are conservative, as the authors only considered the highest risk groups for each disease (25).

### Others

In addition to a reduction in visits for specific RMNCH, HIV/AIDS, or malaria care, attendance at health facilities in general decreased during the outbreak. Two studies in Sierra Leone identified a drop in the performance of surgical care. One study found a 70% drop (*p* = 0.005) in the median number of admissions to the facilities and a 50% decrease (*p* = 0.014) in the median number of major surgeries, mainly hernia repairs and cesarean sections, during the first half of 2014. If the level of care remained as low for the rest of 2014, an estimated 35,000 in need in Sierra Leone would be excluded from inpatient care between mid-May and December 2014 (7). The second study found a reduction of 63–81% in accident and emergency presentations and ward admissions between 2013 and the end of 2014; the volume of operative surgery fell to 3% of the 2013 volume by the end of 2014, and 25% of the surgeons at the facility died from Ebola (13). Visits also decreased in Guinea by 31% at hospitals and 6–40% at health centers in the last 3 months of 2014 (21) and at community health centers in Sierra Leone in the most affected chiefdoms, although no change was seen in visits in unaffected chiefdoms (16).

Finally, a general estimation of the causes of death was modeled to compare the number of EVD deaths to deaths by other leading causes in Guinea, Sierra Leone, and Liberia. In Liberia, the model indicated that EVD deaths exceeded the expected number of deaths from other causes. In Sierra Leone, EVD potentially caused more deaths in 2014 than malaria, which normally is the leading cause of death. In Guinea, EVD caused fewer deaths than the top three non-EVD causes (19).

## Discussion

This review indicates that the magnitude of indirect health effects due to the West Africa Ebola outbreak is substantial, but it is also clear that further studies needs to be done post-disaster to better understand the overall health effects of a crisis of this scale. All studies included in this systematic review show a decrease in health service provision or utilization. Estimates from the various studies suggest both morbidity and mortality to have increased after the onset of EVD in Sierra Leone, Guinea, and Liberia. RMNCH was especially affected and the decrease in facility deliveries, cesarean sections, and volume of ANC and PNC visits is likely to have a significant impact on maternal and newborn health. Emergency obstetric care such as cesarean sections is the most effective mean to lower maternal mortality (33) and the unmet obstetric need in these three countries was very high even before the EVD outbreak (34). A decrease in the already low number of women accessing emergency obstetric care in Sierra Leone, Liberia, and Guinea are likely to cause additional deaths among mothers and newborns.

Even if the adult HIV prevalence is estimated at below 2% in all three countries, with around 20% of infected individuals being on ART, HIV/AIDS remains one of the top 10 causes of mortality. Several studies saw an increase in both ART defaulters and admission of new patients. Service provision during this time remained fairly stable, indicating that the decrease in treatment was due to the reluctance among patients to visit health facilities during the EVD outbreak. The authors of one of the studies conclude that during a crisis such as the Ebola outbreak, HIV care is challenging but not impossible (21). However, the EVD outbreak is likely to have a long-term impact on the burden of HIV/AIDS in these countries, due to disrupted treatment and a potential increase of infectiousness as well as a delayed onset of treatment for newly infected people.

Malaria was a substantial burden in Sierra Leone, Guinea, and Liberia before the EVD outbreak. The studies included in this review clearly show that a disruption in both preventative and curative measures for malaria would increase morbidity and mortality. For instance, IPT in pregnancy has repeatedly been shown to decrease cases of malaria amongst pregnant women, which in turn prevents the adverse effects of malaria in pregnancy, including low birth weight and neonatal deaths (35). Several studies in this review showed a substantial decrease in cases of malaria seen at health facilities after the onset of the EVD outbreak, increasing the risk of severe and cerebral malaria, especially in children. However, malaria care was maintained during the outbreak at a greater extent than expected in some of the models.

This review shows a decrease in vaccination coverage for pentavalent vaccines in Guinea and Sierra Leone, and the potential effect of a disruption in measles vaccination programs. For countries with low vaccination coverage before the outbreak, the effects could be more severe. The suspension of vaccine programs during the outbreak will have long-standing adverse consequences, including the potential for a higher rate of transmission of vaccine-preventable diseases and a lower proportion of fully vaccinated children. Making large-scale nationwide vaccination campaigns a main priority in the recovery phase for these three countries could mitigate adverse outcomes.

By May 2015, 1.45% of Guinea’s doctors, nurses, and midwives had died of Ebola; in Liberia, 8.07% died and 6.85% died in Sierra Leone. In countries where the number of health workers was already low, the deaths are sure to have a lasting impact on population health, as is highlighted in a recent policy paper by Evans et al. (17, 32). Here, the authors estimate that approximately 25,000 additional deaths per year could occur due to the death of health-care workers.

Interestingly, a study on obstetric health care in Sierra Leone showed that those facilities that remained open during the outbreak continued to perform the same proportion of both deliveries and cesarean sections as they did before the Ebola outbreak. This suggests that the nationwide decrease in deliveries and cesarean sections is due to the closure of key facilities, or in some cases, the conversion of hospitals into Ebola Treatment Units. The same study also found that government hospitals were able to maintain their health service provision to a much larger extent than both non-profit and for-profit private facilities (8). It is clear that many health-care workers kept working despite the harsh and frightening conditions at the time of the outbreak. Importantly, this review shows that to a large extent, it was not health service provision that failed, but rather the uptake of health services by the population that decreased after the onset of the Ebola outbreak. Not all studies suggested reasons for this, but references have been made to the fear of nosocomial transmission of the disease and mistrust of authorities. Health centers and hospitals became centers of disease transmission and by ignoring cultural traditions and beliefs, mistrust increased. Furthermore, service uptake may have decreased as a result of misinformation on whether facilities were open and providing services during the outbreak. However, it is not possible to identify a cause for of the decreases from the results of this review, and it is likely that disruption of services also played a role in the decline in service delivery. Although no conclusions can be drawn on equity in access to health care during the EVD outbreak, it is plausible that both access and uptake of health services was differentially affected depending on where in the country people reside.

### Strengths and Limitations of the Study

The EVD outbreak is still recent and, as of yet, there is not a substantial body of evidence on the subject. It is likely that there is a publication bias in this area of research toward adverse health effects and impacts of the EVD outbreak, or a worsening of the situation. It remains to be seen whether the EVD outbreak had some beneficial impact on health systems of the affected countries, perhaps by increasing their resilience and capacity to withstand future crises. The quality of studies included in the review varied. Due to the nature of the EVD outbreak, many studies were rapidly initiated and opportunistic, rather than comprehensive and carefully planned. Because of the opportunistic approach used in many of the studies, it is not possible to draw conclusions yet on the actual relationships and causes between the outcomes and the outbreak. Since health-care facilities were considered one of the main sources of infection, data collection had to be done very cautiously. One result of this is the use in the included studies of already available, routine data, perhaps leading to the overrepresentation of findings for RMNCH, HIV/AIDS, or surgical procedures, since visits for these are commonly tracked and recorded in all three countries.

In the modeling studies, it was assumed that service was disrupted for months, which in hindsight was not the case. However, these “worst case scenario” estimates are useful as a planning tool for future interventions for future outbreaks. The non-peer-reviewed reports included in this review were found to be unbiased and were based on robust data, collected using valid scientific methods.

There is possibly an overlap between some of the studies, where they have used the same source of data. Studies also used different time points in defining pre- and post-Ebola, meaning that study results must be carefully applied outside the context they were studied in. Furthermore, it is not possible to give a comprehensive overview of the effects in each country, due to the limited number of studies on similar outcomes across the three countries.

Besides these limitations, the strength of this study lies in including all relevant studies on indirect health and health service effects of the West Africa EVD outbreak up until March 2016. Two coauthors of this study have direct field experience from working in affected countries in the midst of the outbreak, both with patient treatment and with policy development and intervention planning.

## Conclusion

Understanding the damaging effect the West Africa Ebola outbreak had on health and health services in the affected countries can help guide policy development on health system strengthening and hopefully increase their resilience for withstanding future shocks. At this early point after the end of the EVD epidemic, the full magnitude of the indirect effects is yet to be understood and documented. However, it can be seen already that the adverse impact of Ebola stretches far beyond Ebola cases and deaths. This review indicates that indirect effects can be long term and highlights the importance of international support to routine health service delivery and the maintenance of vaccination programs and preventative and curative malaria programs, both in general but especially in times of emergencies. The most important response to an outbreak of this magnitude comes now, before the next outbreak – when we can learn from past mistakes and build back stronger and more resilient health systems.

## Author Contributions

Conception or design of the work: KR and DS. Data collection: KR and DS. Data analysis and interpretation: KR and DS. Drafting the article: KR. Critical revision of the article: KR, DS, AE, and JS. Final approval of the version to be published: KR, DS, AE, and JS.

## Conflict of Interest Statement

The authors declare that the research was conducted in the absence of any commercial or financial relationships that could be construed as a potential conflict of interest.
